# Semi-automatic carotid intraplaque hemorrhage detection and quantification on Magnetization-Prepared Rapid Acquisition Gradient-Echo (MP-RAGE) with optimized threshold selection

**DOI:** 10.1186/s12968-016-0260-3

**Published:** 2016-07-16

**Authors:** Jin Liu, Niranjan Balu, Daniel S. Hippe, Marina S. Ferguson, Vanesa Martinez-Malo, J. Kevin DeMarco, David C. Zhu, Hideki Ota, Jie Sun, Dongxiang Xu, William S. Kerwin, Thomas S. Hatsukami, Chun Yuan

**Affiliations:** University of Washington, Seattle, WA USA; Walter Reed National Military Medical Center, Bethesda, MD USA; Michigan State University, East Lansing, MI USA; Tohoku University, Miyagi, Japan

**Keywords:** Intraplaque hemorrhage, Carotid atherosclerosis, Semi-automatic quantification, CMR, Threshold, MP-RAGE

## Abstract

**Background:**

Intraplaque hemorrhage (IPH) is associated with atherosclerosis progression and subsequent cardiovascular events. We sought to develop a semi-automatic method with an optimized threshold for carotid IPH detection and quantification on MP-RAGE images using matched histology as the gold standard.

**Methods:**

Fourteen patients scheduled for carotid endarterectomy underwent 3D MP-RAGE cardiovascular magnetic resonance (CMR) preoperatively. Presence and area of IPH were recorded using histology. Presence and area of IPH were also recorded on CMR based on intensity thresholding using three references for intensity normalization: the sternocleidomastoid muscle (SCM), the adjacent muscle and the automatically generated local median value. The optimized intensity thresholds were obtained by maximizing the Youden’s index for IPH detection. Using leave-one-out cross validation, the sensitivity and specificity for IPH detection based on our proposed semi-automatic method and the agreement with histology on IPH area quantification were evaluated.

**Results:**

The optimized intensity thresholds for IPH detection were 1.0 times the SCM intensity, 1.6 times the adjacent muscle intensity and 2.2 times the median intensity. Using the semi-automatic method with the optimized intensity threshold, the following IPH detection and quantification performance was obtained: sensitivities up to 59, 68 and 80 %; specificities up to 85, 74 and 79 %; Pearson’s correlation coefficients (IPH area measurement) up to 0.76, 0.93 and 0.90, respectively, using SCM, the adjacent muscle and the local median value for intensity normalization, after heavily calcified and small IPH were excluded.

**Conclusions:**

A semi-automatic method with good performance on IPH detection and quantification can be obtained in MP-RAGE CMR, using an optimized intensity threshold comparing to the adjacent muscle. The automatically generated reference of local median value provides comparable performance and may be particularly useful for developing automatic classifiers. Use of the SCM intensity as reference is not recommended without coil sensitivity correction when surface coils are used.

## Background

Intraplaque hemorrhage (IPH) is an important plaque feature that is associated with accelerated plaque progression [1] and increased risk of plaque rupture [2]. In both previously asymptomatic [1] and symptomatic patients [3], the presence of IPH by cardiovascular magnetic resonance (CMR) has been shown to be predictive of future ischemic events.

CMR is a noninvasive imaging modality that has been shown to be capable of detecting IPH, attributed to the T1 shortening effect of methemoglobin [4]. Magnetization-prepared rapid acquisition gradient echo (MP-RAGE) is a highly T1-weighted sequence and has been widely used for IPH detection, with the advantages of good tissue contrast, high spatial resolution and short scan time. MP-RAGE was first applied for the identification of IPH at 1.5T [5] and further implemented at 3.0T [6]. At 3T, MP-RAGE has been shown to outperform T1-weighted fast spin-echo and time-of-flight sequences in detecting IPH [7].

In most previous studies, hemorrhage was identified manually as hyperintense signal by visual inspection or using an arbitrary threshold of 1.5 to 2.0 as compared to the signal intensity of normal arterial wall or the adjacent muscle (Table 1) [3, 7–10]. However, manual quantification of IPH is problematic as small IPH and the peripheral areas of large IPH are often less distinct and may suffer from poor inter-reviewer reproducibility with subjective criteria. To our knowledge, studies to determine the optimum objective criteria for IPH detection in MP-RAGE with histology validation are lacking. Furthermore, recent serial studies have indicated that dynamic changes of vessel wall hyperintense signals exist and may be associated with plaque progression [11–14]. Therefore, automatic techniques that allow reliable IPH detection and quantification with histology validation are needed for understanding the clinical implications of hyperintense signal evolution within atherosclerotic plaques.

**Table 1:** Previous studies that identified IPH (or high signal intensity) using MP-RAGE

Study	No of subjects	Histology	Threshold	Reference tissue	Field Intensity (T)	Coil	TR/TI (ms)	Comments
Yamada et al. [9]	222	No	2.0	Adjacent muscle	1.5	Standard neck array and spine array coil	1500/660	Define high signal intensity regions
Hishikawa et al. [8]	35	Yes	2.0	Adjacent muscle	1.5	Standard neck array and spine array coil	1500/660	Define high signal intensity regions
Altaf et al. [3]	64	No	1.5	SCM	1.5	Receive-only quadrature neck array cervical spine coil	1462/741^a^	Define regions for IPH
Mendes et al. [10]	35	No	1.5	SCM	3	Custom designed 4 or 16 element phased array surface coils	667/370	Define regions for potential IPH
Ota et al. [7]	20	Yes	Subjective	Normal artery wall or adjacent muscle	3	Four-channel phased-array surface coil	568/304	Manually define regions for IPH

*IPH* intraplaque hemorrhage, *MP*-*RAGE* magnetization-prepared rapid acquisition gradient-echo, *SCM* sternocleidomastoid muscle

^a^Calculated based on imaging parameters reported in the paper

The aim of this study was to develop a semi-automatic method for IPH detection and quantification based on the widely available MP-RAGE sequence. The method was optimized by comparing multiple tissue references and determining the hyperintensity threshold that afforded the highest diagnostic accuracy using histology as the gold standard.

## Methods

### Study population

A total of 14 patients (10 men, 4 women; mean age, 69 years ± 12 [standard deviation]) scheduled for carotid endarterectomy were recruited for this study. Among them, 4 patients were symptomatic and 11 patients had greater than 80 % carotid stenosis. This study was compliant with the Declaration of Helsinki and was approved by the institutional review boards of Ingham Cardiothoracic & Vascular Surgeons (Lansing, MI). All patients provided written informed consent.

### MR imaging protocol

Patients were imaged on a 3T Signa HDx MR scanner (GE Healthcare, Waukesha, WI) using a dedicated 4-channel carotid bilateral phased-array coils (Pathway MRI, Seattle, WA) prior to carotid endarterectomy. A previously described 3D MP-RAGE sequence was used [6]: TR/TE = 13.2 ms/3.2 ms, flip angle = 15°, in plan spatial resolution = 0.63 mm × 0.63 mm, reconstructed resolution = 0.31 mm × 0.31 mm, slice thickness = 1 mm, number of excitation = 2, TI = 304 ms, TR with respect to the nonselective inversion = 568 ms, acquisition time = 3 min 50 s. Fat suppression was achieved by water selective excitation.

### Histology

The histology protocol for tissue processing and staining has been previously described [7, 15, 16]. Briefly, carotid endarterectomy specimens were excised en bloc, fixed in formalin and decalcified in 10 % formic acid. The whole specimen was then embedded in paraffin and sectioned (10 μm) at every 1.0 mm in the common carotid artery and at every 0.5 mm in the internal carotid artery. Sections were stained with hematoxylin-eosin and Mallory’s trichrome. Digital photographs of slides were taken. Pixel size on the histology images were calibrated by photographing a calibration grid of known size. Regions containing erythrocytes and hemorrhagic debris (red to brown on Mallory’s stain) were identified and outlined in the digital images using a custom segmentation program (MATLAB, R2010b, US) by an experienced histologist (M.S.F.) blinded to CMR. Areas of IPH regions by histology were calculated based on the outlined contours and calibrated pixel size. Scattered intact erythrocytes without tissue structure disruption or inflammatory response were considered artifacts. Presence or absence and area of IPH were recorded for each histology section.

Matching between histological sections and MR images was done based on the relative distance to carotid bifurcation. Lumen and outer wall morphology and calcifications provided confirmatory information for satisfactory matching. Because of the difference in slice thickness, one MR image may have several matched histological sections, in which case the averaged IPH area of matched histological sections was calculated and IPH was considered present if it was identified in any of the matched histological sections.

### IPH detection and quantification on CMR

#### Reference selection

In the literature, IPH was described as being hyperintense on T1-weighted images such as MP-RAGE. Since the original pixel values in MP-RAGE images vary not only between patients, but also between axial slices due to the inhomogeneous coil sensitivity, the original pixel values should be normalized by dividing the signal intensity of a reference region. The choice of a reference to calculate the threshold can affect diagnostic performance.

**Fig. 1:**
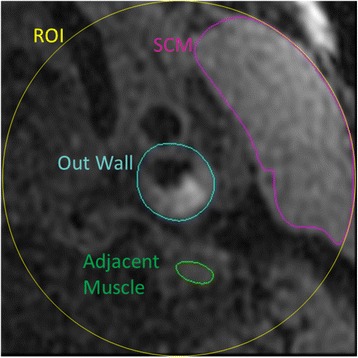
MP-RAGE image slice with reference contours. The reference region was confined by an ROI (*yellow circle*) centered at the carotid artery with a diameter of 4 cm, while the IPH detection area was confined by the out wall boundary (*blue*). The three references were 1) mean intensity of SCM (sternocleidomastoid muscle, pink), 2) mean intensity of adjacent muscle (*green*) isointense with the normal carotid artery wall, and 3) median intensity within the ROI

Three tissue references were evaluated in developing semi-automatic IPH characterization (Fig. 1): 1) the mean signal intensity of sternocleidomastoid muscle (SCM), calculated from the portion that was within a 4 cm diameter circular region of interest (ROI) centered at the carotid lumen to reduce the influence of signal inhomogeneity associated with surface coil; 2) the mean signal intensity of adjacent muscle where an ROI was drawn on the nearest muscle (such as the scalene) with the similar signal intensity as normal carotid artery wall; and 3) the median signal intensity within the 4 cm circular ROI which has been used as a reference in previous studies for signal normalization in automatic plaque segmentation [17, 18].

The first two references needed manually drawn ROI while the third one was more automatically generated. The adjacent muscle reference was chosen beside the SCM because the SCM was closer to the skin surface and may be biased towards a higher value relative to the carotid artery.

#### Threshold selection

Receiver operating characteristic (ROC) curves for IPH detection were generated for each of the reference methods. The overall performance was summarized using the area under the ROC curve (AUC) (MATLAB, R2010b, US). The optimal thresholds were then chosen as those which maximized the Youden’s index (sensitivity + specificity-1).

Given the difference in spatial resolution between histology and CMR, the size of IPH could have an impact on in vivo IPH detection. Furthermore, it is common to observe colocalization of IPH and calcification during histological assessment, which could affect the signal intensity of IPH because of magnetic susceptibility effect on gradient echo [7, 16]. Therefore, in this study, all the analyses were conducted using six data subsets, respectively: 1) all slices, 2) subset excluding slices with small IPH (area <1.25 mm^2^ as measured on histology), 3) subset excluding slices with small IPH (area <2.8 mm^2^), and 4) – 6) subsets with the same criteria of 1) -3), respectively, except that slices with heavily calcified IPH (>50 % IPH area calcified) were also excluded. The small IPH area cutoff values were determined using π(0.63x)^2^ with x being 1 and 1.5, respectively, where 0.63 (mm) was the resolution of MP-RAGE images [7].

#### IPH detection and quantification

To confine the area for IPH detection and measurement, loose out wall boundaries that enclosed the carotid artery were drawn by experienced reviewers. All pixels whose signal intensity exceeded the hyperintensity threshold defined by the tissue reference were outlined as IPH regions (MATLAB, R2010b, US). The presence/absence and the area of IPH in each MP-RAGE slice were recorded.

#### Cross-validation

Leave-one-out cross-validation was used to test the robustness of the semi-automatic method [18]. Specifically, images of each subject were analyzed using the semi-automatic method based on the optimized thresholds derived from the other subjects. By comparing with histology gold standard, sensitivity and specificity for IPH detection and Pearson’s correlation coefficient for IPH area quantification were calculated (MATLAB, R2010b, US).

## Results

133 axial MR slices from 14 patients were matched with histology specimens, among which 63 (47 %) slices had IPH identified on histology. Eight of the 133 slices were excluded when the SCM was used as reference, because the SCM in those slices were more than 2 cm away from the artery, outside the 4 cm diameter circular ROI.

**Fig. 2:**
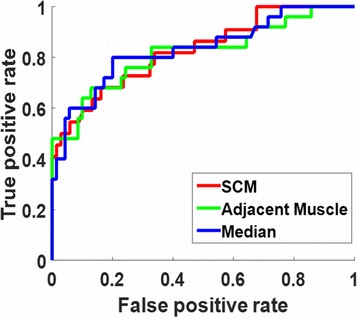
Receiver operator characteristic curves using three references (SCM, adjacent muscle and median intensity). The subset of slices without heavily calcified IPH or small IPH of area smaller than 2.8 mm^2^ was used

ROC curves for the subset excluding slices with IPH area <2.80 mm^2^ or heavily calcified IPH are plotted in Fig. 2. The AUC for each subset is shown in Table 2 for all three references. When compared to the full dataset, the AUC increased when heavily calcified IPH and IPH with area <2.80 mm^2^ were excluded. There were no significant differences observed for the AUC between different references.

**Table 2:** The optimized thresholds for detecting IPH

Data subset			AUC			Optimized threshold		
	IPH Area (mm^2^)^a^	n+/n^b^	SCM	Adjacent muscle	Median	SCM	Adjacent muscle	Median
With CA	>0	63/133	0.69	0.71	0.58	1.0	1.6	2.2
	>1.25	52/122	0.71	0.72	0.64	1.0	1.6	2.2
	>2.80	34/104	0.74	0.79	0.68	1.0	1.6	2.2
No CA^c^	>0	49/119	0.74	0.71	0.66	1.0	1.7	2.2
	>1.25	40/110	0.76	0.71	0.72	1.0	1.7	2.2
	>2.80	25/95	0.83	0.80	0.80	1.0	1.6	2.2

*AUC* area under the ROC curve, *IPH* intraplaque hemorrhage, *ROC* Receiver operating characteristic, *SCM* sternocleidomastoid muscle

^a^IPH area: excluding IPH with areas < π (0.63x)^2^, where 0.63 mm was the in-plane resolution of MP-RAGE, and x was set as 1 and 1.5 which gave areas of 1.25 and 2.80

^b^n+: number of slices with IPH present in histology; n:total number of slices

^c^No CA: excluding heavily (>50 %) calcified IPH; with CA: not excluding heavily calcified IPH

The optimized thresholds to detect the presence of IPH are summarized in Table 2. The optimized IPH signal intensity thresholds varied little across different data subsets and remained at 1.0 times the SCM intensity, 1.6-1.7 times the adjacent muscle intensity and 2.2 times the median intensity when slices with small IPH or heavily calcified slices were included or excluded.

**Fig. 3:**
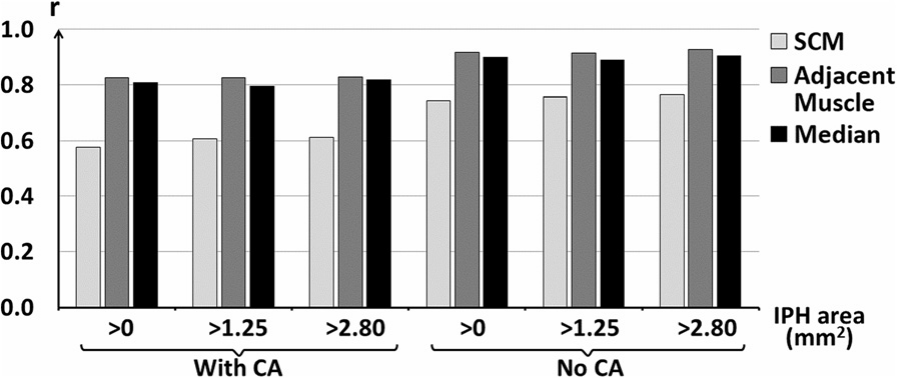
Pearson’s correlation coefficients (r) of IPH area measured in MP-RAGE images and histology. IPH areas were detected using the optimized thresholds based on three kinds of reference for different subsets of data, respectively

**Fig. 4:**
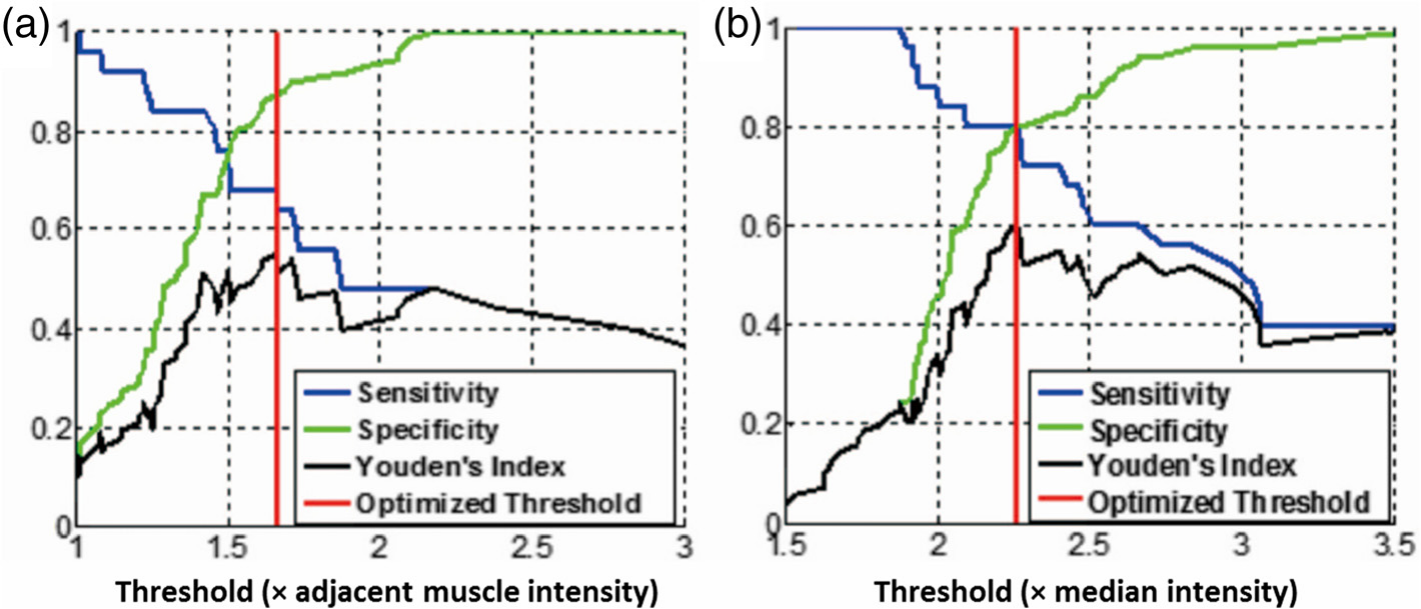
Sensitivity and specificity using different thresholds based on two types of references. (**a**) The adjacent muscle and (**b**) the median intensity within the ROI. Red line shows the optimized thresholds (heavily calcified IPH areas and IPH area < 2.80 mm^2^ excluded, *n* = 95)

**Fig. 5:**
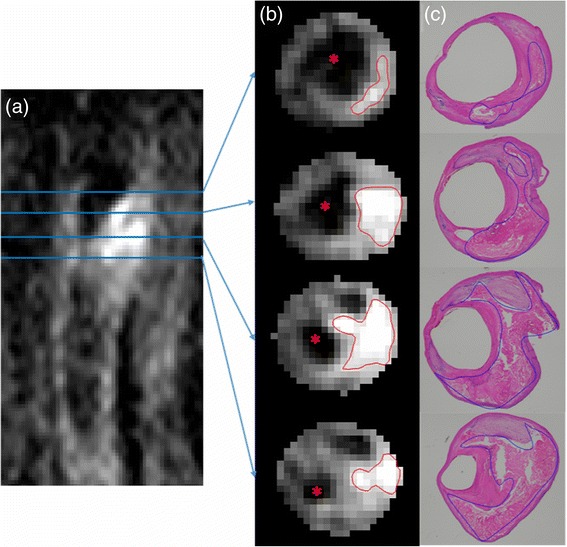
Matched MP-RAGE image slices and histology specimens with IPH contours. (**a**) Coronal MP-RAGE slice of the left carotid artery; (**b**) axial MP-RAGE slices within the out wall boundary at the blue line levels in (**a**) with IPH contours (*red line*) generated semi-automatically using 2.2 times the median intensity as the intensity threshold (MATLAB, R2010b, US); (**c**) matched histology specimens with IPH outlined (*blue line*). Red asterisk in (**b**) indicates lumen

IPH detection sensitivity, specificity and IPH area quantification correlation between CMR and histology using the three different references are shown in Table 3. During cross-validation, the optimized thresholds for each reference varied by less than 10 % across each leave-one-out data set. Without excluding heavily calcified IPH and small IPH, the sensitivity of IPH detection was relatively low (sensitivity = 49, 56 and 40 %, respectively, using the SCM, the adjacent muscle or the median as reference). After excluding heavily calcified IPH and small IPH, sensitivity increased to 59, 68 and 80 %, respectively, using the SCM, the adjacent muscle and the median as reference; specificity and Pearson’s correlation coefficients (r) of IPH area between CMR and histology also increased overall (specificity = 85, 74 and 79 %; *r* = 0.76, 0.93 and 0.90, respectively, using the SCM, the adjacent muscle and the median as reference). IPH area correlation based on the adjacent muscle and the median were relatively higher than that based on the SCM (Fig. 3), although there were no statistically significant differences between them. The sensitivity, specificity and Youden’s index for various thresholds using the adjacent muscle and the median as reference are shown in Fig. 4. Semi-automatic IPH segmentation using a threshold based on the median method compared well with the IPH region identified based on histology (one example is shown in Fig. 5).

**Table 3:** IPH detection and quantification performance using the optimized thresholds

Data subset			Sensitivity (%)			Specificity (%)			Pearson’s correlation coefficient (r)		
	IPH Area (mm^2^)	n+/n	SCM	Adjacent muscle	Median	SCM	Adjacent muscle	Median	SCM	Adjacent muscle	Median
With CA	>0	63/133	49	56	40	62	69	80	0.58	0.83	0.81
	>1.25	52/122	57	62	52	76	69	80	0.61	0.82	0.80
	>2.80	34/104	50	68	59	85	70	80	0.61	0.83	0.82
No CA	>0	49/119	54	49	39	66	76	81	0.74	0.92	0.90
	>1.25	40/110	62	53	58	76	74	80	0.76	0.91	0.89
	>2.80	25/95	59	68	80	85	74	79	0.76	0.93	0.90

*IPH* intraplaque hemorrhage, *SCM* sternocleidomastoid muscle

## Discussion

This study demonstrated that the semi-automatic detection and quantification of carotid intraplaque hemorrhage on MP-RAGE CMR can be obtained by optimizing thresholds with proper references using histology as the gold standard. The choice of reference for signal normalization affected the optimized threshold and IPH detection performance.

A threshold of 1.6 based on the adjacent muscle intensity was found to have good performance for IPH detection with high correlation (*r* = 0.83-0.93) for IPH area quantification. As such, the adjacent muscle method is recommended for IPH detection where an adjacent muscle region that is isointense with the normal artery wall can be readily identified and manually outlined. For a more automatic IPH detection method, the local median method appears to be the optimal choice for efficiency and accuracy, considering that the threshold of 2.2 based on the median intensity also has good IPH detection performance with IPH area correlation *r* = 0.81-0.90. The optimized threshold for the commonly used the SCM reference method [3, 10] was around 1.0, due to the vicinity of the SCM with the surface coils. Also, the measured IPH area correlation between MP-RAGE and histology was relatively low using the SCM as reference compared with the adjacent muscle and the median, although these differences were not statistically significant, potentially due to our limited sample size. Therefore, using the SCM as reference may be problematic without coil intensity correction when surface coils are used, where the SCM has elevated signal intensity. Thus use of the adjacent muscle or the median method is recommended over the SCM.

Compared with manual IPH detection, semi-automatic IPH detection has comparable performance, yet it has the advantage of higher repeatability and requires less time. Ota et al. [7] manually outlined IPH for 20 patients scheduled for carotid endarterectomy using the MP-RAGE sequence and evaluated the performance by referring to histology ground truth. Using the semi-automatic median technique, we found better IPH area correlation with histology in the present study than in Ota’s study (*r* = 0.90 compared with *r* = 0.81), comparable sensitivity (80 % in both), but lower specificity (79 % compared with 97 %). It should be noted that manual segmentation reproducibility is affected by the inconspicuous boundaries of IPH and window level settings, which is further aggravated by the lack of objective criteria on intensity threshold. Of the limited number of studies which reported on manual measurement of IPH area, Touze et al. [19] found that the intraobserver and interobserver reproducibility on IPH area quantification were suboptimal (ICC = 0.70 and 0.60, respectively). The semi-automatic method can theoretically achieve near-perfect reading reproducibility if using the automatically generated local median intensity as reference. Nonetheless, interscan reproducibility remains to be evaluated. Using the adjacent muscle as reference introduces additional variability than using local median intensity. Despite the limited signal-to-noise of MP-RAGE, to find such a reference was usually not challenging as the goal was to sample a region that had similar signal intensity as normal carotid wall rather than to accurately identify the anatomy. Additionally, manual segmentation of IPH areas can be time-consuming given the increasing use of high-resolution protocols that result in big volumes of images. In a recent study [20], IPH plaques had an average length of 19.4 mm. Given the MP-RAGE resolution (1 mm slice thickness) used in this study, manual segmentation would take 20–30 min depending on image quality and reviewers’ experience. In contrast, the semi-automatic method does not require detailed segmentation of vessel wall or reference tissue, and would take about 5 min using the adjacent muscle as reference and 2–3 min using automatically generated local median intensity as reference.

Figure 4 provides thresholds for selection by determining the desired optimum sensitivity and specificity for the adjacent muscle and the median methods. A threshold with higher sensitivity may be preferred to screen patients for IPH, whereas a threshold with a lower false positive rate may be preferable for clinical decision-making.

Furthermore, our study demonstrated the limitation of MP-RAGE CMR sequence in detecting heavily calcified IPH and small IPH. This is likely due to 1) co-localized calcification resulting in lowered IPH signal intensity and 2) IPH areas smaller than the detection threshold of MP-RAGE. Sensitivity, AUC and Pearson’s correlation coefficient progressively increased when heavily calcified IPH and/or IPH smaller than cutoff area were excluded. However, the optimal threshold was found to be stable regardless of the presence/absence of these characteristics. This suggests that using these thresholds can detect IPH with the caveat that sensitivity may be reduced in populations with small or heavily calcified IPH. This limitation is inherent to CMR given the limited resolution (compared to histology) that is afforded by current techniques. Nonetheless, in previous studies, CMR-detected IPH, which presumably corresponds to large IPH in histology, has been found to be associated with plaque progression and future cardiovascular disease [1, 3], and larger CMR-depicted IPH area has been associated with recent [21] and subsequent events [22]. Therefore, the limitations in detecting small IPH and heavily calcified IPH do not appear to preclude CMR from being a meaningful tool for detecting clinically significant IPH. The proposed method provides semi-automatic IPH quantification and may facilitate future studies on the clinical significance of IPH size.

It is possible that variations among scanners, sequence parameters or use of different coils may cause signal ratio variance from those reported here. Choice of receiver coil can have a big impact on IPH/muscle signal ratio, as shown in the present study. Dedicated carotid coils use surface arrays to improve local signal reception, which has been increasingly used in carotid CMR studies. However, receive coil sensitivity for such surface coils decreases from the surface of the skin towards the center of the neck. Tissues closer to the skin such as SCM tends to have higher signal than deeper tissues such as the carotid artery. Different algorithms exist for surface coil intensity correction [23, 24] and correcting the signal drop-off may partially correct for the receive coil sensitivity difference. However, this effect is difficult to quantify and may differ between coil geometries and correction algorithms used. Furthermore, many carotid CMR studies do not use coil intensity correction. As such, our results comparing different references provide a practical guidance for clinicians and researchers who perform carotid CMR studies on IPH. Since the adjacent muscle reference is outlined in a region very close to the carotid artery with similar signal intensity as the normal artery wall, and the local median intensity is based on the ROI with a diameter of 4 cm centered at the carotid artery, differences in coil sensitivity will affect the adjacent muscle and the median reference less than the SCM. Use of coil intensity correction may improve performance of SCM relative to adjacent muscle. Sequence parameters such as inversion time (TI) and repetition time between successive inversion pulses can also affect signal ratios. Therefore, we used MP-RAGE sequence parameters recommended as optimum by Zhu et al. [6] at 3T. Different scanner platforms may also differ in implementation details such as type of inversion pulse used, reconstruction filters etc. Such scanner platform differences may cause minor signal ratio variance. To account for these unavoidable variances, a phantom with multiple T1 values mimicking muscle and IPH might be useful for signal intensity calibration.

### Limitations of the study

One limitation of the current study is that the thresholds were optimized based on IPH presence/absence in each slice, without considering the location of detected IPH within the slice or its size. Nevertheless, based on our observation, the location of detected IPH in MP-RAGE mostly coincided with the true IPH location in histology when checked visually (Fig. 5). While the optimized threshold was determined without consideration of size of IPH, our study demonstrates that the size of IPH measurement using the semi-automatic method correlated well with that in histology (*r* = 0.93 for the adjacent muscle and *r* = 0.90 for the median). In addition, due to our small sample size, we were not able to test the performance of each threshold on an independent sample, though we applied cross-validation to reduce the bias associated with optimizing and testing thresholds using the same data set. The semi-automatic method was developed based on the widely used MP-RAGE technique. There are other emerging techniques, but they have not become widely available. It is conceivable that T1-mapping would allow more accurate measurements of IPH. Techniques that can perform atherosclerosis T1-mapping with sufficient image quality and clinically acceptable scan time warrant further investigations.

## Conclusions

With histology validation, a semi-automatic intraplaque hemorrhage detection and quantification method based on an optimized threshold was developed. Use of the adjacent muscle as reference is recommended where an adjacent muscle region isointense with the normal carotid artery wall can be manually defined; while for a more automatic IPH detection, reference of the local median intensity is recommended. The sternocleidomastoid muscle is not recommended as a reference when surface coils are used without coil sensitivity correction.

## Abbreviations

AUC, area under the ROC curve; CA, calcification; CMR, Cardiovascular magnetic resonance; IPH, intraplaque hemorrhage; MP-RAGE, Magnetization-Prepared Rapid Acquisition Gradient-Echo; ROC, Receiver operating characteristic; SCM, sternocleidomastoid muscle
